# Evaluation of Antibiotic Prophylaxis and Discharge Prescriptions in the General Surgery Department

**DOI:** 10.7759/cureus.4793

**Published:** 2019-06-01

**Authors:** Cem Karaali, Mustafa Emiroğlu, Bülent Çalık, ısmaıl Sert, Eyup Kebapci, Tayfun Kaya, Gokcen G Budak, Gökhan Akbulut, Cengiz Aydın

**Affiliations:** 1 General Surgery, University of Health Sciences Tepecik Training and Research Hospital, Izmir, TUR; 2 Infectious Diseases, Sakarya University, Sakarya, TUR

**Keywords:** surgical antibiotic prophylaxis, inappropriate discharge prescription, questionnaire

## Abstract

Introduction

Although there are international guidelines for surgical antibiotic prophylaxis (SP), the use of inappropriate SP is still a common problem. Most studies investigated SP applications in clean and clean-contaminated cases. However, antibiotics in the discharge prescriptions of these cases have not been adequately investigated. In this study, we aimed to examine the antibiotics in SP applications and discharged prescriptions together and to find out the causes of inappropriate use.

Materials and methods

We retrospectively evaluated the data of patients admitted to our general surgery wards between 2014 and 2015. Patients with clean or clean-contaminated wound category operations were included. The patients were evaluated in terms of convenience of SP (choice of antibiotics, compliance with an indication for SP, timing of the first dose, SP>24 hours, and discharge prescription). In addition, to interpret the results, a questionnaire has been performed for the surgeons in the same clinics.

Results

A total of 1205 patients with clean and clean-contaminated wound class operation were enrolled in this study. The total accuracy rate of SP was 7.1%. SP application with the correct indication and timing of the first dose was compatible with guidelines: 55.6% and 81.9%, respectively. SP was applied >24 hours at 60.2% and antibiotic prescribing carried out after discharge at 80.6% of patients. According to questionnaire results, the use of SP over 24 hours and the prescription of antibiotics during discharge were: drain usage, hyperthermia, leukocytosis, surgeons feeling of comfort, avoidance of patients, and their relatives' reactions.

Conclusion

The total accuracy rate of SP rate was low in the present study and in surgeons prescribing the SP after discharge. In light of the present study, we suggest that discharge prescriptions should also be reviewed in clinics who have a high inappropriate surgical antibiotic prophylaxis rate.

## Introduction

Surgical antibiotic prophylaxis (SP) aims to reduce postoperative surgical site infections (SSIs). If an SSI develops as a postoperative complication, the length of hospital stay, readmission rate, and related costs increase [1]. Therefore, proper antibiotic prophylaxis is essential. For proper antibiotic prophylaxis, guidelines state that an antibiotic should be administered within one hour before the surgical incision, the correct antimicrobial agent should be given, and the duration of usage should not exceed 24 hours [2]. However, when the literature was examined, it has been shown that compliance with the surgical antibiotic prophylaxis guidelines is frequently not closely followed [3-5]. Due to the overuse of antibiotics and other drugs, there is a current policy toward "rational drug use" in Turkey, as in other parts of the globe, and substantial successful progress has been achieved [6]. However, certain studies on surgical antimicrobial prophylaxis report accuracy rates of less than 20% at all stages of appropriate prophylaxis [7-9]. While previous studies have investigated the surgical prophylaxis (SP) applications in our country [3,9], our study simultaneously evaluates SP administration, discharge prescriptions, and a questionnaire administered in the research and training hospital.

The aim of the present study was to evaluate the appropriateness of the antibiotic prophylaxis that applied in the department of general surgery and doctor’s reasons that affect the prophylaxis. This would help us identify priorities for improving the implementation of surgical antibiotic prophylaxis as part of our country's "rational drug use" policies.

## Materials and methods

Patients who underwent surgery at Izmir Tepecik Training and Research Hospital, General Surgery Clinic, in 2014-2015 were enrolled in the study. The study was approved by the local ethics committee of Izmir Tepecik Training and Research Hospital. Data were obtained retrospectively from the electronic database of patients with clean and clean-contaminated wound class operations. These group operations were defined as thyroidectomy, inguinal, incisional, or umbilical hernia repair with mesh, breast cancer surgery, elective cholecystectomy, gastrectomy, and colorectal operations. The timing of administration, duration, and choice of antibiotics were evaluated. The suitability of the SP was determined according to the surgical antibiotic prophylaxis guidelines also used in our clinic [2]. In addition, we reviewed the antibiotics added to discharge prescriptions as a continuation of SP. In order to determine the reasons for inappropriate prophylaxis, a questionnaire was performed on the surgeons practicing in our clinics.

Certain cases were excluded from the study: emergency operations, cases where sterile conditions had not been maintained, and patients undergoing early cholecystectomy for acute cholecystitis. Surgeons who refused to fill in the questionnaire were also excluded from the study.

Statistics

The statistical program SPSS for Windows 22.0 (Statistical Package for the Social Sciences Inc., Chicago, IL, USA) was used to evaluate the data. Descriptive statistics are presented as numbers and percentages for categorical variables and as mean and standard deviation for numerical variables.

## Results

A total of 1205 patients were included in the study; 545 (45.2%) patients were male and 660 (54.8%) were female. The mean age of the patients was 50 ± 9.25. The most commonly performed operation was laparoscopic cholecystectomy (Table 1). Cefazolin was the most commonly used antibiotic (84.9%); others used were ampicillin-sulbactam (13%), ciprofloxacin (1%), and ceftriaxone and tigecycline (1%).

The total accuracy of SP, in terms of indications, time of administration of preoperative prophylaxis, duration of administration of antibiotics, was appropriate according to the guidelines in 141 (11.7%) cases. When we examine the discharge prescription with SP together, the total accuracy of SP falls to 7.1%. Compliance with the indication for SP preoperatively, according to guidelines, was compatible in 671 (55.6%) cases. This is presented in detail in Table 1.

**Table 1:** Application of surgical antibiotic prophylaxis (SP) preoperatively according to operations *Compliance with guideline

Surgery type	SP applied n (%)	SP not applied n (%)	Total n (%)
Laparoscopic cholecystectomy (low risk)	318 (92.2%)	27* (7.8%)	345 (100%)
Open cholecystectomy + high-risk laparoscopic cholecystectomy	84* (100%)	0 (0%)	84 (100%)
Thyroidectomy	161 (83%)	33* (7%)	194 (100%)
Mastectomy	122* (89.7%)	14 (10.3%)	136 (100%)
Hernia repairs	264* (86.5%)	41 (13.5%)	305 (100%)
Gastric operations	75* (100%)	0 (0%)	75 (100%)
Colorectal operations	66* (100%)	0 (0%)	66 (100%)
Total	1090 (%90.4)	115 (%9.6)	1205 (%100)

In 986 (81.9%) cases, antibiotic prophylaxis was administered within one hour prior to surgical incision but in 219 patients (18.1%), this was delayed until during or after the operation. In addition, 725 (60.1%) patients were treated with antibiotic prophylaxis in the postoperative period for longer than 24 hours and 971 patients (80.6%) were treated with antibiotic prescription during discharge (Tables 2-3).

**Table 2:** Prophylaxis times according to operation

Surgery type				
	SP<24 hours n (%)		SP>24 hours n (%)	Total n (%)
Laparoscopic cholecystectomy (low risk)	200 (58%)		145 (42%)	345 (100%)
Open cholecystectomy and high-risk laparoscopic cholecystectomy	0 (0%)		84 (100%)	84 (100%)
Thyroidectomy	144 (74.2%)		50 (25.8%)	194 (100%)
Mastectomy	91 (66.9%)		64 (33.1%)	136 (100%)
Hernia repair	41 (13.4%)		264 (86.6%)	305 (100%)
Gastric operations	3 (4%)		72 (96%)	75 (100%)
Colorectal operations	1 (1.5%)		65 (98.5%)	66 (100%)
Total	480 (39.8%)		725 (60.1%)	1205 (100%)

**Table 3:** Antibiotic prescription during discharge

Surgery type	Yes n (%)	No n (%)	Total n (%)
Cholecystectomy	387 (90.2%)	42 (9.8%)	429 (100%)
Thyroidectomy	100 (51.5%)	94 (48.5%)	194 (100%)
Mastectomy	97 (71.3%)	39 (28.7%)	136 (100%)
Hernia repair	272 (89.2%)	33 (10.8%)	305 (100%)
Gastric operations	59 (78.7%)	16 (21.3%)	75 (100%)
Colorectal operations	56 (84.8%)	10 (15.2%)	66 (100%)
Total	971 (80.6%)	234 (19.4%)	1205 (100%)

For evaluating the SP applications, the questionnaire was given to 23 surgeons. Nineteen surgeons completed the questionnaire. According to the questionnaire results, the use of SP over 24 hours and the prescription of antibiotics during discharge were due to drain usage, hyperthermia, leukocytosis, surgeons' feeling of comfort, and avoidance of patients and their relative’s reactions. Table 4 shows the reasons for the prolonged administration of antibiotics. Also, we determined that in the daily routine of our general surgery clinic, all patients were transferred to the operation theater with antibiotics.

**Table 4:** Questionnaire results; the reasons for SP over 24 hrs and the prescription of antibiotics at discharge SP: surgical antibiotic prophylaxis; WBC: white blood cell Participants chose more than one option.

	Clean wound category n (%)	Clean-contaminated wound category n (%)
I do not prescribe	5 (26.3%)	3 (15.8%)
Presence of drain	9 (47.3%)	11 (57.9%)
Fever	9 (47.3%)	9 (47.3%)
Increase of WBC	7 (36.8%)	8 (42.1%)
Major defect of sterility	4 (21.0%)	5 (26.3%)
Feeling more confident	6 (31.5%)	5 (26.3%)
Using as a defense factor in case of developing an infection	5 (26.3%)	5 (26.3%)
Presence of a catheter	6 (31.5%)	9 (47.4%)
Other	4 (21.0%)	5 (26.3%)

## Discussion

In surgical antibiotic prophylaxis, the administration of appropriate agent and timing prior to the operation and cessation beyond 24 hours after the operation is known to be beneficial [2,10]. Several guidelines were presented for surgical clinical applications. Unfortunately, in spite of these guidelines, the inappropriate application of SP continues [3,11-12]. In the present study, the total compliance rates of SP were lower than in other studies in our country (15.4%-19.7%, respectively) [9,13] as well as in Greece [14] and French [15] studies (36.3%-41.1%, respectively). The present study shows the absence of consensus on SP among the general surgeons, and this behavior should be improved. SP for suitable patients may help decrease the rate of surgical site infections, hospital stay, and costs [10]. In addition, SP has positive effects on sepsis-associated mortality [10]. Inappropriate antibiotic usage may cause the development of bacterial resistance and an increase in patient costs [1] and C. difficile-associated colitis [16]. Consequently, appropriate SP is vital for all patients and effective health systems. The rate of unnecessary preoperative prophylactic antibiotic use was higher than those in the literature (39.7% as compared to 11%-19%) [1,14,17]. On the other hand, the avoidance of giving preoperative prophylactic antibiotics, though needed, was lower than in literature (4.5% vs 33%) [2]. According to questionnaire results, surgeons avoided any conflict with patients or their relatives, as reported in the study of Lu et al., by giving SP for even thyroidectomy operations [11]. The other reason for the inappropriate administration of antibiotic is that, in our daily routine, antibiotics are transferred to operating theaters along with the patients and anesthesiologists administer these antibiotics routinely. The increased rates of inappropriate antibiotics use in the present study have originated from this routine.

The present study showed that surgeons used a high proportion of suitable agents in SP. The ideal prophylactic antibiotic should be a low-cost agent with a narrow spectrum and few side effects while reaching a high concentration in surgical wound tissues. Cefazolin meets all these criteria and is an agent that is often recommended by many guidelines [2]. We also found out that cefazolin was the most commonly used antibiotic (84.9%), as stated in another multicenter study [18]. In addition, broad-spectrum antibiotics, such as ceftriaxone and tigecycline, were used inappropriately for prophylaxis in our study. Surgeons may have opted for wider-spectrum antibiotics than narrow-spectrum antibiotics to ensure adequate protection.

SP was highly administered at appropriate times in the present study. One of the components of surgical antibiotic prophylaxis is the appropriate time of the first administration. This is an important concern, as delayed prophylactic antibiotic use can increase surgical site infections [2,19-20]. In our study, 81.9% of antibiotics were started at the right time. A similar result was found in a Malaysian study (80.5%) [21] and a higher result than Italian studies (53.4% and 76%, respectively) [8,22]. On the other hand, 18.1% of the antibiotics were given inappropriately in our study. In order to decrease the error rates further, it has been suggested that the responsibility for administering the SP should be given to just one team member, ideally, the anesthesiologist [17]. These may be the reasons for the administration of SP at the appropriate time in the present study. Apart from this positive effect, the anesthesiology team may be one of the causes of the inappropriate administration of SP.

The present study shows that there are many cases who were given SP for more than 24 hours, and this further affected the prescription of antibiotics after discharge. Although the literature is focused on SP over 24 hours in clean and clean-contaminated wounds, the prescription of antibiotics at discharge is an important issue. Prolonged prophylaxis does not have a positive effect on the patient and it causes superinfections, drug intoxications, and bacteria resistance [1-2]. In the present study, more than half of the patients are administered SP over 24 hours. In addition, the prescription of antibiotics at discharge was 80.6%. Urgancı et al. had similar results for the prescriptions of antibiotics at discharge at a rate of 88.5% [3]. On the other hand, it was found to be higher than the studies of Bozkurt et al. [9] and Choi et al. [23] in which other surgical branches were also examined (17.4%-60.3%, respectively). However, the necessity of ceasing SP after a 24-hour period is very clear for patients with clean and clean-contaminated wound classes [2]. According to our questionnaire results, surgeons declared the reasons for administering SP over 24 hours, as follows: the presence of a drain, fever, increased white blood cell counts, surgeons' feeling of confidence, and avoidance of unnecessary discussions with patients and relatives. The prolonged administration of antibiotics may cause a false sense of confidence. But guidelines do not recommend prolonged antibiotic administration even in the presence of drains [2]. Also, fever and the increase of white blood cell counts are not indications of the prolonged administration of antibiotics. In these conditions, source control and the investigation of other foci of infection should be the case [24]. Interestingly, some studies have found a significant relationship between the cultural dimensions of a country as described by Geert Hofstede [25] and SP>24 hours [26-27]. According to Geert Hofstede, drug prescribers who come from cultures with high uncertainty avoidance (UA) feel uncomfortable with uncertainty and ambiguity and are more likely to prescribe antibiotics when faced with unclear clinical scenarios [28]. In Australia, a positive correlation was found between a high UA and SP>24 hours [27]. The UA score of our country is also high [29]; the role played by cultural factors in the higher than usual proportion of patients being given unnecessary SP or receiving it for over 24 hours seems worth considering. However, no study has been conducted on this issue in our country. As a result, informative meetings should be held for surgeons' concerns.

Limitations

There are certain limitations to our study, including its retrospective nature. In addition, as the study only reflects the attitudes and behaviors of the general surgery clinic in one hospital, care should be taken while generalizing our results to all general surgery clinics.

## Conclusions

In conclusion, this data shows that despite current guidelines, SP are still being administered incorrectly. Even though the agents and time of administration of SP are appropriate according to guidelines, unnecessary SP is determined to be high. For most patients, SP had been administered over 24 hours, and this approach reflected the prescription at discharge. In light of the present study, we suggest that surgeons should review their SP behavior and clinics that have a high inappropriate SP rate should also review discharge prescriptions.
