# Correction: Evaluation of Antibiotic Prophylaxis and Discharge Prescriptions in the General Surgery Department

**DOI:** 10.7759/cureus.c23

**Published:** 2019-08-05

**Authors:** Cem Karaali, Mustafa Emiroğlu, Bülent Çalık, ısmaıl Sert, Eyup Kebapci, Tayfun Kaya, Gokcen G Budak, Gökhan Akbulut, Cengiz Aydın

**Affiliations:** 1 General Surgery, University of Health Sciences Tepecik Training and Research Hospital, Izmir, TUR; 2 Infectious Diseases, Sakarya University, Sakarya, TUR

Due to a calculation error made by the authors, the total accuracy rate was incorrectly stated to be 7.1% when in fact the correct rate is 8%. This change has been made in the following areas within the article:

Abstract, results: "The total accuracy rate of SP was 7.1%." has been changed to "The total accuracy rate of SP was 8%."

Results, second paragraph: "When we examine the discharge prescription with SP together, the total accuracy of SP falls to 7.1%." has been changed to "When we examine the discharge prescription with SP together, the total accuracy of SP falls to 8%."

 

